# Revolutionizing CAR T-Cell Therapies: Innovations in Genetic Engineering and Manufacturing to Enhance Efficacy and Accessibility

**DOI:** 10.3390/ijms251910365

**Published:** 2024-09-26

**Authors:** Lorenzo Giorgioni, Alessandra Ambrosone, Maria Francesca Cometa, Anna Laura Salvati, Robert Nisticò, Armando Magrelli

**Affiliations:** 1 Faculty of Physiology and Pharmacology “V. Erspamer”, Sapienza Università di Roma, 00185 Rome, Italy; lorenzo.giorgioni@uniroma1.it; 2 National Center for Drug Research and Evaluation, Istituto Superiore di Sanità, 00161 Rome, Italy; alessandra.ambrosone@guest.iss.it (A.A.); mariafrancesca.cometa@iss.it (M.F.C.); 3 Faculty of Pharmacy, Tor Vergata University of Rome, 00133 Rome, Italyrobert.nistico@gmail.com (R.N.); 4 Agenzia Italiana del Farmaco, Via del Tritone 181, 00187 Rome, Italy

**Keywords:** CAR T, gene editing, GMP-in-a-box, ATMP manufacturing, hospital exemption

## Abstract

Chimeric antigen receptor (CAR) T-cell therapy has achieved notable success in treating hematological cancers but faces significant challenges in solid-tumor treatment and overall efficacy. Key limitations include T-cell exhaustion, tumor relapse, immunosuppressive tumor microenvironments (TME), immunogenicity, and antigen heterogeneity. To address these issues, various genetic engineering strategies have been proposed. Approaches such as overexpression of transcription factors or metabolic armoring and dynamic CAR regulation are being explored to improve CAR T-cell function and safety. Other efforts to improve CAR T-cell efficacy in solid tumors include targeting novel antigens or developing alternative strategies to address antigen diversity. Despite the promising preclinical results of these solutions, challenges remain in translating CAR T-cell therapies to the clinic to enable economically viable access to these transformative medicines. The efficiency and scalability of autologous CAR T-cell therapy production are hindered by traditional, manual processes which are costly, time-consuming, and prone to variability and contamination. These high-cost, time-intensive processes have complex quality-control requirements. Recent advancements suggest that smaller, decentralized solutions such as microbioreactors and automated point-of-care systems could improve production efficiency, reduce costs, and shorten manufacturing timelines, especially when coupled with innovative manufacturing methods such as transposons and lipid nanoparticles. Future advancements may include harmonized consumables and AI-enabled technologies, which promise to streamline manufacturing, reduce costs, and enhance production quality.

## 1. Introduction

All currently approved CAR T cells are autologous products, where cells are used as starting material for the medicinal product derived from a patient, making each batch a personalized product. The use of unique starting materials entails a high level of variability among batches of the “same” product, making this manufacturing approach harder to optimize than that of traditional medicinal products, substantially increasing costs and thereby limiting sustainable and widespread access. Moreover, despite being at the forefront of care in several oncologic indications, these therapies are curative in a minority of cases, and struggle when translated in the solid tumor indication. The main limitations of CAR T-cell therapy in this field consist of the presence of the TME and its immunosuppressive resident immune cells, as well as the heterogeneity of solid tumors. The TME is a hypoxic environment with poor nutrient supply, making it difficult for T cells to persist and function in it. It also restricts the trafficking of CAR T cells because of its dense extracellular matrix and abnormal angiogenesis [1], presenting a physical barrier that is able to prevent sufficient infiltration, limiting the ability of CAR T cells to reach tumor cells effectively. The TME is rich in immunosuppressive cells like tumor-associated macrophages (TAMs), which inhibit the activity of CAR T cells by interacting with them through immunosuppressive signals, such as cytokines, and immune-checkpoint ligands such as PD-L1 [2] Because solid tumors are highly heterogeneous with different cell populations, the antigen expression within the same solid mass is diverse. This variability means that only a portion of a tumor will be effectively targeted by CAR T cells. Moreover, even when targeted, tumor cells can downregulate or lose their antigen, thus developing resistance to CAR T-cell therapy [3].

This review addresses these limitations by exploring ways to make access more economically viable, specifically dealing with new manufacturing methods and new machinery to implement those methods. A focus is provided on compact and automated systems, known as “closed systems”, which may address shortfalls in the production of autologous products. Such shortfalls, like long manufacturing times and high costs, are addressed in the review’s respective sections. New research results on synthetic biology and genetic engineering are reported as feasible alternative approaches to overcome the limitations in the efficacy of current therapies in solid tumor indications, thus making CAR-mediated therapies more widely applicable. Among the plethora of solutions being investigated in the current landscape of CAR T research, those reported here are characterized by their general scope of application, which should make them easier and, therefore, more likely to be incorporated in future developments of CAR T-cell therapy. 

## 2. Enhancing CAR T-Cell Therapy Efficacy

### 2.1. Genetic Engineering of T Cells as a Potential Solution to Lack of Persistent Action In Vivo

Two critical issues faced by the current generation of CAR T-cell therapies are immunogenicity and their tendency to get exhausted. CAR T-cell therapy’s immunogenicity is a significant challenge, as the immune system can recognize the engineered T cells as foreign and mount a response against them. A significant approach to mitigate this issue involves engineering the transmembrane domain of CAR T cells.

This domain is responsible for anchoring the CAR to the engineered T-cell’s membrane [4]. This mediates the stability and, therefore, the functioning of CAR constructs, influencing crucial activities of CAR T cells, such as activation-induced cell death (AICD) [5]. By modifying it, the immunogenicity of CAR T cells could be reduced. This could be achieved using transmembrane domains from human proteins such as CD8 or IgG4 to replace murine elements with humanized sequences to decrease the CAR’s recognition as non-self by the host immune system [6]. This would make the CAR less likely to be targeted and destroyed by the immune system, allowing it to function longer and more effectively, and this would be compounded by the increased stability. The phenomenon of CAR T exhaustion is being actively researched [7,8] because of its impact on the efficacy of the medical product over the long term. In the case of cancer treatment, CAR T exhaustion is accompanied by a new proliferation of tumor cells. Different research groups have explored methods to rejuvenate these cells, particularly through genome alterations such as the activation or deactivation of specific genes with the objective of boosting CAR T cells and influencing their activity. By mimicking the gene activity of stem cells, this approach aims to preserve the ability of CAR Ts to kill cancerous cells for longer periods. This constitutes a new avenue of research to surpass one of the main limitations of CAR T cells [9,10]; instead of optimizing the CAR moiety, the whole T cell would be altered to multiply more quickly and last longer in patients, ultimately leading to increases in the safety and efficacy of future CAR T-cell versions.

#### 2.1.1. CAR T-Overexpressing FOXO1

A recent study [11] compared different samples of CAR T cells targeting leukemia to detect cellular proteins regulating their gene activity. A set of genes more active in fit CAR T cells was identified, all regulated by a master-switch protein called FOXO1. This protein was instead found to be depleted in CAR Ts coming from recipients who did not respond well to treatment. The same researchers conducted additional studies by modifying CAR T cells to increase FOXO1 production and then administering these altered cells into a mouse model. Using unaltered CAR T cells as a control group, they found that the modified cells were more effective in reducing tumor size in mice and demonstrated greater longevity in the body compared to the standard CAR T cells. A similar phenomenon was also observed by a different team examining the effect of interleukin-15 (IL-15) when administered alongside CAR T cells in clinical trials [12]. By examining the gene activity in CAR T cells exposed to IL-15, higher levels of FOXO1 were found to be associated with activated cells. This group also engineered CAR T cells to produce higher levels of FOXO1. If the effectiveness of this approach is confirmed in clinical trials, engineering CAR T cells to overexpress FOXO1 might become a possible new manufacturing step to be considered in future productions.

#### 2.1.2. CAR T-Overexpressing SUV39H1

Jain and colleagues investigated the maintenance of CAR T therapeutic benefits by preventing their exhaustion, focusing on the effects of a gene called SUV39H1 [13], which encodes the enzyme histon-lysine-N-methyltransferase, which is involved in the methylation process of proteins. CAR Ts overexpressing this gene were produced and evaluated in mice models of leukemia and prostate cancer, where the edit avoided relapse and led to remission. Inhibiting the expression of SUV39H1 seems to prevent the activation of various genes implied in T-cell longevity, thereby resulting in the control and regulation of many other genes simultaneously. Additional clinical investigations are needed to determine the efficacy of this discovery.

Other engineering approaches have also been reported [14], focusing on reducing the number of CAR T cells and increasing their longevity without compromising their cytotoxic capacity, which is crucial for the treatment’s efficacy. In addition, lowering the dose, like with any medicinal product, would contribute to reducing the overall toxicity of the treatment, that, in the case of CAR Ts, would lead to the minimization of common immune reactions, such as the cytokine release syndrome (CRS). 

#### 2.1.3. CAR T-Overexpressing C-Jun

C-Jun is a member of the AP-1 family of transcription factors, which is required for T-cell activation, whose activity is strongly augmented by TCR signaling through Jun kinase. c-Jun overexpression renders CAR T cells exhaustion-resistant [15]. A c-jun overexpressing CAR T-cell therapy, named LYL797, is currently being investigated in trial NCT05274451. It targets an antigen called ROR1, which is overexpressed in several solid tumor types, including non small cell lung cancer (NSCLC) and triple-negative breast cancer (TNBC). Overexpression of c-Jun is expected to induce stem-like characteristics to T-cell populations, hence resulting in a more durable antitumor activity of the CAR T-cell product [16].

### 2.2. Construct Engineering of CARs as a Potential Solution to Lack of Persistent Action In Vivo 

CAR-engineered medicinal products hold great promise but lack durability and are prone to relapse, with durable remissions only observed in a limited number of patients [17]. The factors likely to cause relapse are tumor antigen loss, mediated by cellular events such as antigen escape and trogocytosis, as well as T-cell exhaustion, which results in dysfunction and poor in vivo persistence [18,19]. Exhausted T cells have been characterized [20] as having complete or partial loss of proliferative capacity and effector function, upregulation of multiple immune inhibitory receptors and transcriptional and epigenetic alterations [21,22,23,24]. Improving the clinical efficacy of CAR T-cell therapy thus necessitates the development of new strategies to solve these challenges, by designing more advanced CARs.

#### 2.2.1. Metabolic Armoring with IL-10

The strategy involves genetically modifying CAR T cells to secrete interleukin-10 (IL-10), thus enhancing their metabolic fitness and enabling them to maintain functionality and survival by reversing the associated metabolic and mitochondrial dysfunctions. The latter is particularly impactful, in that it involves mitochondrial depolarization, which leads to the inhibition of oxidative phosphorylation and an increase in oxidative stress. This results in decreased cell activity and higher expression of exhaustion markers such as PD-1 [25,26]. Metabolic armoring can restore mitochondrial fitness, and restart activity in exhausted CAR T-cells by effectively reprogramming their metabolism and altering their transcription [27,28]. The abovementioned approach improved outcomes in various cancer models, as thoroughly reported in a recent Swiss study [29]. IL-10 armoring led to complete regression in established solid and metastatic tumor models across several cancer types in syngeneic and xenograft murine models. IL-10 paracrine signaling also allowed CAR T cells to induce stem cell-like memory responses in lymphoid organs, resulting in durable protection against tumor rechallenge. A potential limitation of this strategy is the association between IL-10 and the initiation of cytokine release syndrome (CRS). This correlation will need to be thoroughly investigated to ensure safety in a clinical setting.

#### 2.2.2. Dynamic CAR Expression via CTLA-4 Tail

Adding cytotoxic T-lymphocyte-associated antigen-4 (CTLA-4) cytoplasmic tails (CCTs) to CARs enables dynamic regulation of their expression by conferring them CTLA-4’s endocytic cycling ability. CTLA-4 is an immune-checkpoint molecule, crucial for maintaining self-tolerance and homeostasis [30]. It cycles between the cell surface and the cytosol owing to its CCT’s YVKM motif, which interacts with clathrin adaptor activating protein 2 (AP-2), resulting in the endocytosis-mediated regulation of surface CTLA-4, for optimal T cell activation [31,32,33]. This outcome is particularly relevant, as it tackles the following major obstacle of all CAR-engineered medicinal products and an unsolved event in current CAR designs: trogocytosis. During this process, lymphocytes extract membrane fragments from antigen-presenting cells (APCs) through the immune synapse and present them on their own surface [34,35,36,37,38]. This leads to fratricide killing which can result in tumor antigen depletion (trogocytosis-induced immune escape) and, consequently, can compromise CAR T efficacy [31,32,33], also leading to exhaustion and uncontrolled inflammatory adverse events. Tailing of CAR Ts does not require generating entirely new CARs for each cancer type [32,33,34,35], and as such this approach provides a unique flexibility. A recent study from Yale [30] reported altering CAR T cells with duplex CTLA-4 CCTs (CAR-2CCT) fusion to their C terminus and then in vitro coculturing with NALM6 cells, a known leukemia model. By controlling the timing and duration of CAR expression, they reported optimized efficacy with limited toxicity; CAR-2CCT was able to completely lyse the leukemia cell line with higher survival rates than control CAR T cell, while expressing less exhaustion markers such as PD-1. These cells were also subjected to repeated stimulation by introducing a second round of NALM6 cells to simulate cancer relapses and exhibited increased survival when compared with control CAR T cells. The study also reported an in vivo test on a relapsed leukemia model, where CAR-CCT cells were able to control leukemia development in NSG mice. Synthetic CCT fusion can reduce continuous CAR activation, decrease trogocytosis, and limit toxicity, thus enhancing CAR’s cytotoxic response upon repeated antigen stimulation.

#### 2.2.3. KITv Signaling for Enhanced Functionality

Providing multiple types of costimulation is the basis of third- and fifth-generation constructs, respectively providing the same type of third stimulatory signal (via a second costimulatory domain) or providing a different third signal (canonically cytokine signaling through JAK–STAT3/5 [39]). To date, these efforts have achieved variable results, hampered by dysfunctional CAR T cells and/or higher cytokine-induced toxicity [40,41]. By integrating KITv, a mutation that allows c-Kit signaling activation independently of its ligand, a fifth-generation CAR construct has been recently reported. c-Kit, also known as CD117, is a type-III receptor tyrosine kinase expressed in stem cell populations and essential for the development of several cell types such as erythrocytes and germ cells [42]. Physiologically, c-Kit signaling is a key driver of T cells’ ability to proliferate and differentiate, though T cells downregulate this pathway during differentiation [43]. The D816V mutation c-Kit’s activating loop (KITv) constitutively triggers c-Kit signaling [44,45,46,47], acting as signal 3 of costimulation and insulating the CAR T cell from immunosuppressing signals within the TME. The construct exhibited remarkable efficacy in tumors with transforming growth factor-β (TGFβ)-induced immunosuppression, as well as low-antigen expression. TGFβ is one of the main immunosuppressive cytokines in solid tumors such as mesothelioma and lung cancer [48,49,50,51]. Another approach to deal with TGFβ that should be briefly mentioned is the use of a dominant-negative transforming growth factor-beta receptor 2 (TGFβRII) (DNTGFβRII), since it has already been reported at the clinical level, demonstrating feasibility [52] DNTGFβRIIs can block TGFβ signaling, so that CAR T cells can remain activated and proliferate effectively. Ideally, c-Kit would surpass the improvement in efficacy resulting from approaches like this, though one of its potential limitations, and the reason why it is downregulated in mature cells, is its correlation with tumorigenesis [53,54,55,56]. which may happen in the case of c-Kit signaling dysregulation/gain-of-function mutations. In the study, KITv CAR T cells reportedly could not proliferate in the absence of antigen activation, supporting the article’s safety claim [57]. Moreover, since c-Kit is a receptor tyrosine kinase, KITv CAR T cells are highly sensitive to market-authorized tyrosine kinase inhibitors (Dasatinib), providing a novel safety switch approach [58].

**Figure 1:**
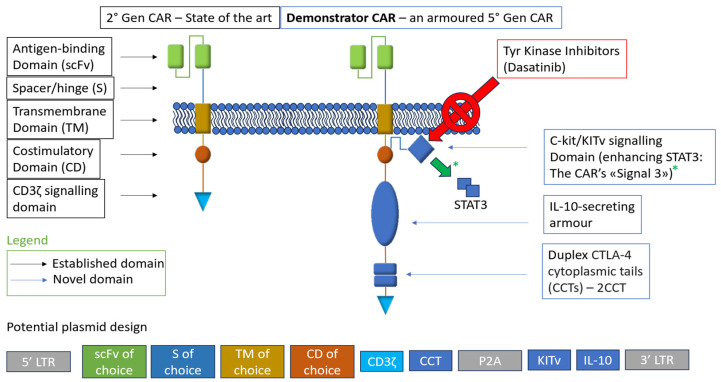
“Demonstrator CAR” is a fictional testbed incorporating all innovations regarding the construct presented in this article.

In principle, these solutions can be integrated into a single construct, possibly incorporating relevant gene edits into an induced pluripotent stem cell (iPSC) platform for unlimited, pre-edited cells to be primed for universal CAR targets, as discussed in the following section. A testbed design, incorporating all innovations discussed above and compared to conventional second-generation construct structural architecture, is represented in Figure 1.

### 2.3. Construct Engineering of Cars as a Potential Solution to Lack of Efficacy in Solid Tumor Indications: Efficacy through Targeting Regardless of Tumor Type

Clinical trials with CAR T-cell therapies against solid tumors have so far yielded disappointing results. One recent meta-analysis on the subject reported only a 9% overall response rate (ORR) across 22 clinical trials [59]. This limited response stems from several factors, including immunosuppression within the TME and the heterogeneous expression of tumor antigens on healthy tissues, leading to poor specificity, treatment resistance and on-target off-tumor toxicity [60,61]. Such inefficient homing of CAR T- cells is responsible for tumor escape, resulting in patient relapse [62]. The vitality of cellular starting material is another factor limiting CAR T efficacy. This issue is due to the current way of sourcing the starting materials to manufacture living drugs. Future possible solutions include allogeneic sourcing from healthy donors and use of non-T immune-type cells or T-cell banks resulting from the use of iPSC technology. The former approach’s limitations are due to the selection of the target antigen(s), which ideally should be found only on malignant cells. Unfortunately, the expression of tumor antigens is heterogeneous and may happen also in healthy tissues. The canonical target of most authorized products, CD19, is an example of this heterogeneity; CD19 is expressed on both healthy and malignant B cells, causing on-target off-tumor cytotoxicity like B cell hypoplasia. While that side effect can be managed, targeting healthy cells in noncancerous tissues risks organ damage, leading to organ failure and potentially death. Furthermore, a single tumor mass will contain subpopulations with different antigens, resulting in escape and positive selection of all tumoral subpopulations without the targeted antigen. The study of solid cancer-specific antigens remains limited, highlighting a significant knowledge gap that needs to be addressed. Research is essential to identify and validate optimal tumor antigens as cancer-specific CAR T targets or to develop alternative strategies for directing CAR T cells against solid tumors. Currently, both types of targets used in CARs show limitations, as follows: overexpressed self-antigens, such as human epidermal growth factor receptor 2 (HER2), are often expressed in healthy tissues, causing on-target off-tumor toxicities [63]. On the other hand, mutated cell surface proteins (neoantigens), like epidermal growth factor receptor III (EGFRvIII), are restricted to cancer cells but can be downregulated or heterogeneously expressed among tumor subpopulations, leading to resistance and positive selection of antigen-negative cancer cells [64]. Targets with wide applicability across different tumor types provide an additional way to lower costs and improve accessibility. If the CAR is effective in treating multiple cancers, economies of scale will make the costs and accessibility of a few products more feasible. Examples of advances against solid tumors involve combinatorial approaches, such as CARs that recognize many antigens [39] or can interact with antigen-low tumor cells; the main examples of the former category are three different variations in dual CARs. The co-administration approach involves the use of two separate CAR T-cell products, each targeting a different antigen and administered sequentially as a combination therapy, with the aim of enhancing the overall efficacy by targeting multiple cancer markers simultaneously [65]. In bicistronic CARs, a single CAR T cell is engineered to express two different CARs from one vector, allowing for the simultaneous targeting of two antigens. Ideally, this would reduce the likelihood of cancer cells escaping detection [66]. Lastly, tandem or bivalent CAR T cells are designed with a single CAR structure that has two antigen-binding domains, enabling the CAR T cell to bind to two different antigens at the same time, improving specificity and, thus, safety, as well as reducing the risk of antigen escape [67]. These innovative strategies aim to improve the effectiveness and durability of CAR T-cell therapies.

However, these approaches are intrinsically limited by the complexity of safely fine tuning their expression to maintain effectiveness. This complexity increases with each additional antigen and is further complicated by the varied antigen levels across patients and tumor types. An alternative strategy would be to discover targets that are either more widely expressed or more effective. Widely expressed targets on solid tumor cells include B7-H3 and nfP2X7, while amph-ligands represent a potentially effective tumor-agnostic method.

#### 2.3.1. Nanobody-Targeting B7H3 as Antigen-Binding Domain

In solid tumors, B7 homolog 3 protein (B7-H3) transmembrane protein [68], also known as CD276, is an emerging immunotherapy target, particularly appealing for its overexpression in multiple solid tumor types, including hepatocellular carcinoma (HCC), lung adenocarcinoma (LUAD), and neuroblastoma (NB) [69], while mostly absent in healthy patients. In humans, two isoforms of B7-H3 have been reported, though knowledge about the function(s) of these two isoforms remains limited [68]. More than 20 clinical trials, including CAR T therapy [70,71] targeting B7-H3, are in progress. An American study from the NCI used B7-H3 CARs engineered with single-chain variable fragments (scFvs) and demonstrated encouraging antitumor activity and/or acceptable safety in preclinical studies against hematologic and multiple solid tumor types, including unconventional scFvs such as dromedary camel nanobodies. B7-H3 CAR T-cells are characterized by highly activated T-cell signaling and significant tumor infiltration. The study used single-cell transcriptome RNA sequencing coupled with functional T-cell proteomics analysis to highlight the upregulated genes involved in persistence of CAR T-cells in their mice models.

#### 2.3.2. Scfv-Targeting nfP2X7 as Antigen-Binding Domain

nfP2X7 is a dysfunctional conformation of the P2X purinoceptor 7 (P2X7), an ATP-gated cation-selective channel controlling ion transport in response to ATP binding. Physiologically, it allows for Na^+^ and Ca^2+^ influx and K^+^ efflux driving downstream signaling pathways involved in cell survival and proliferation, though under prolonged binding to ATP under ATP-rich conditions, P2X7 fulfils the opposite activity by acting as a nonselective pore, permeable to large molecules (<900 Da), resulting in apoptosis [72]. While P2X7 is widely expressed in many tissues [73], the channel adopts the nfP2X7 conformation only in malignant cells, where it exposes a unique epitope normally buried in its internal structure. Antibodies recognizing this epitope have been developed, revealing significant overexpression of nfP2X7 on many solid tumors, including brain, breast, lung, pancreas, and stomach. In this conformationally constrained nonfunctional form, the channel retains its ion transporter function but cannot mediate apoptosis [74]. This is thought to allow nfP2X7-positive cells to avoid apoptotic cell death in ATP-rich conditions, such as the TME, conferring them a selective advantage. While the in vitro data presented in the study are attractive, their translation in vivo presents limitations. In xenograft mouse models of breast and prostate cancers, CAR T-cells targeting nfP2X7 did not cure the mice, being only able to manage tumor growth for a short period of time, and the tumors themselves had no time to form a TME, as CAR T administration occurred only 6 days after the inoculation of tumors. Both pitfalls could be due to researchers accounting for the difficulty CAR T-cells face in trafficking and infiltrating the solid tumor mass. The discovery of targets like these, despite the highlighted limitations, may redefine cancer targeting by setting a precedent that broadly expressed tags exist in nature, common to most tumors.

#### 2.3.3. ScFv Amph-Ligand Target as Antigen-Binding Domain

A recent approach for the development of CARs able to deal with downregulation and antigen escape or lack of tumor-specific antigens was reported. The tumor-agnostic antigen-binding domain concept is based on the insertion of intra-tumorally (i.t.) administered exogenous ligands on the membranes of target cells, which can then bind to a specific CAR, thus working independently of antigen expression on the target tissue. The strategy has already been reported, for instance, using oncolytic viruses to transduce tumor cells with antigens such as CD19 [75]. These previous attempts suffered the heterogeneity and highly variable transduction achieved by viral vectors both within individual tumors and among patients [76,77]. The highlighted research, instead, reported a phospholipid–polymer conjugate that, when delivered locally via i.t. injection, labels tumor cells with a small-molecule antigen and tags them for destruction by T cells with a CAR targeting the small-molecule antigen known as fluorescein isothiocyanate (FITC), which makes an amphiphile tag when conjugated with lipid–poly(ethylene)-glycol (PEG) and can insert its lipid tail into the plasma membrane of cells [78]. Via this “amphiphile tagging”, CAR T-cells may be redirected against any solid tumor mass. Following systemic infusion, CAR T-cells targeting the ligand can effectively traffic to, accumulate, and proliferate inside the tumor. Moreover, these amph-ligands decorate antigen-presenting cells (APCs) [79] via “antigen or epitope spreading” [80], recruiting the host’s immune system to address metastases. In different in vivo syngeneic murine solid tumor models, the construct was able to halt tumor progression for several weeks and extend survival of treated mice. The safety of the method was assessed by analyzing the potential leakage of amph-FITC outside the tumor into surrounding healthy tissue by immunohistochemistry on adjacent nontumor tissue, revealing sparse uptake of amph-FITC on cells in the neighboring healthy tissues. Potential systemic toxicities were also evaluated without significant results. Repeated i.t. doses of amph-FITC were reported as target boosting, and the importance of intra-tumoral dosing frequency was assessed. A drawback of this approach is the need for local injection of amph-ligand, as it limits the application of the therapy to accessible tumors [81]. With the development of additional i.t. therapies, the technologies used for the administration of these agents (such as image-guided injections) will likely be applied more widely [82].

## 3. CAR T-Cell Therapy Manufacturing: Limits and Future Perspectives

### 3.1. Limitations of Modern-Day ATMP Manufacturing, Including Car T: Long Production Times and High Costs

The currently authorized process for engineering T cells is costly, complex, time-intensive, and hard to scale up, resulting in a production rate that falls short of meeting the medical demand across all approved indications. A key factor driving up costs is the personalized nature of the treatment; because cells and other biologic materials vary among individuals, standardizing the requirements of starting and raw materials is particularly challenging. The quality of starting materials obtained from a patient depends on a multitude of factors, including their age and illness, making each production run unique. In addition, to ensure the highest rate of production success, raw and starting materials, as well as reagents, need to be subject to extensive and long-lasting quality controls, fully characterized [83], and provide assurance that they are free from communicable agents, such as adventitious agents, viruses, and transmissible spongiform encephalopathies (TSEs).

The sophisticated genetic engineering necessary to modify cells with the chimeric antigen receptors (CARs), as well as the extended time required for cell expansion to ensure an adequate dose and adhere to good manufacturing practice (GMP) standards, with rigorous quality-control testing for safety, potency, purity, and sterility, coupled with comprehensive release testing to confirm a product’s suitability for infusion, and concluding with complex logistics for handling and transporting cells under adequate conditions, all of these factors contribute to the overall complex and time-intensive nature of cell-based manufacturing processes. This prevents the adoption of a conventional industrial approach for small-molecule production and demands significant resources in terms of both funds and time, which are critical factors for patients.

The time required for manufacturing is widely considered one of the therapy’s significant limitations, as it contributes to the inability of some patients to receive treatment despite having successfully undergone the first step of this medical procedure, which is the removal of white blood cells (i.e., leukapheresis). This issue is consistently observed across clinical trials, where the rapid progression of the disease often leads to patient mortality before they can receive the drug product. For instance, the time it takes to manufacture and perform release testing of one authorized CAR T medicinal product, which is now becoming an established option in the hands of hematologists, usually takes about 3 to 4 weeks [84], which may result in becoming incompatible with the life expectancy of the receiving patient.

Addressing the slow manufacturing process could reduce the need for difficult bridging therapies, such as radiotherapy, which can further harm patients (and are often not very effective), while also increasing the percentage of patients who receive treatment with their engineered cells.

Being cell-based products, all ATMPs must be produced under strictly controlled manufacturing conditions that ensure adherence to good manufacturing practices (GMPs) for sterile medical products. This includes the setup of adequate facilities, with proper control over the environmental conditions, furnished with suitable equipment and dedicated instruments operated by qualified personnel, who must have access to high-quality materials and reagents (e.g., culture media) and perform delicate and lengthy quality control operations to ensure the overall compliance of the products. Running these facilities is extremely expensive in terms of financial and human resources, not only during the setup and construction phases but also for their maintenance and the day-to-day operational costs. These costs are reflected in the price of advanced therapy products and limit this therapeutic option to large clinical centers. Since the development of many ATMPs originates in academic or research institutions affiliated with hospitals, these costs also affect research efforts. The final price for the patient includes research and development expenses, as well as compensation for past clinical trial costs, losses from failed production runs, and the profit margins of the commercializing company.

**Figure 2:**
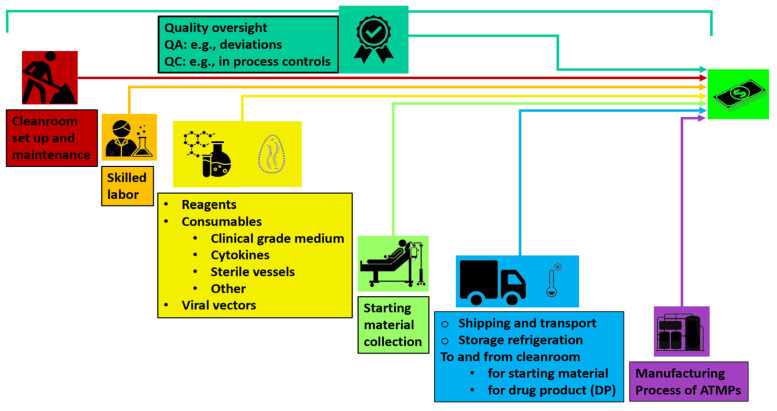
The factors that result in the cost of ATMPs today. Quality oversight is reported on top because it encompasses all other aspects of setting up and running a traditional cleanroom operation.

The factors making up the crude cost of ATMPs are shown in Figure 2.

Reducing costs and production time is thus key to expanding access to CAR T-cell therapy, enabling more patients to receive treatment and smaller centers to offer it. Recent research highlights the role of cell health in therapy efficacy, suggesting earlier treatment could improve outcomes. Streamlining these processes could boost clinical research and accelerate the adoption of CAR T-cell therapies in mainstream treatment.

#### Lowering Car T-Cell Therapy’s Cost to Improve Access and Expand Clinical Trials

Since their approval in the latter half of the 2010s, CAR T therapies in western countries have been priced beyond the reach of most individuals, and healthcare systems cannot scale their deployment. While these costs for the national health systems often include highly skilled professionals, leukapheresis, and shipping to and from centralized cell-manufacturing facilities, they do not consider hospital fees, ICU admissions, and side-effect treatments. This cost barrier limits the wider adoption of CAR T therapies and may hinder trials that compare them to the standard of care for potential future first-line use.

In the 2020s, there has been increased competition to develop less expensive processes in the autologous CAR T space. Emerging economies, such as India, Brazil, and Thailand, have developed and received approval for their own versions of these ex vivo gene therapies, with significantly lower costs than western counterparts. Investigating these cost reductions could help translate similar efficiencies to western markets, making CAR T-cell therapies more sustainable and accessible globally. For instance, the Indian company ImmunoACT completed a phase 2 clinical trial for actalycabtagene autoleucel (actaly-cel) in 50 patients with B-cell malignancies [85], with a projected production cost of USD ~15,000 per patient, at the scale of 300 patients/year, compared to approximately USD 300,000 to 500,000 of currently authorized CAR T therapies. Actaly-cel therapy received marketing authorization from the local national regulatory authority (India’s Central Drugs Standard Control Organization) [86] in October 2023, under the name NexCar19 [87]. Although not currently evaluated by other authorities, such as the EMA, if its manufacturing meets international requirements and GMP standards, it will raise significant questions about the pricing structure in western countries. Another Indian developer named Immuneel also reported results for a phase II study on another construct, Varnimcabtagene autoleucel [88], with similar costs.

Many components of CAR T-cell production, such as the anti-CD19 CAR T vector and some manufacturing platforms, including automated devices used for cell selection and processing, can be commercially purchased, facilitating validation and reducing costs by enabling onsite production at the point of care. This avoids expensive shipping and reduces costs, while ensuring GMP compliance and critical quality controls, which is necessary for maintaining patient safety.

The use of starting materials from healthy donors (to manufacture allogeneic products) could be considered a valid alternative to cut costs, as it offers greater consistency, standardization, and improved manufacturing times, potentially enhancing the safety profile. Allogeneic products would more closely resemble traditional biologics in their development with respect to characterization, specifications, release, and stability testing before administration. These “off-the-shelf” therapies, like other medicinal products, eliminate shipping delays since they are ready in advance. Allogeneic CAR T therapies are gaining traction, with some now being used under hospital exemption protocols. However, the allogeneic CAR T technology cannot be considered fully mature yet, as it equally presents a significant set of challenges, the most important being the decreased efficacy with respect to their autologous counterparts. Achieving the right balance between decentralized manufacturing and stringent quality controls will be key to making CAR T-cell therapy more affordable and accessible worldwide, while maintaining safety standards.

### 3.2. Newer Manufacturing Methods

#### 3.2.1. Transposons as the Heir to Retro- and Lenti-Viral Transduction

CAR T-cells engineered using the Sleeping Beauty (SB) transposon system have demonstrated promising antileukemic activity while minimizing severe toxicities [89]. The Sleeping Beauty system, which integrates the CAR gene into T-cell genomes, offers an alternative to viral-based methods, which are the state of the art for generating CAR T cells at scale. This system’s advantages include a lower risk of insertional mutagenesis, simpler and potentially safer production, reduced immunogenicity, and cheaper manufacturing versus viral vector manufacturing costs in terms of quality controls, since fewer assays are needed for their qualification. The engineered CAR T-cells using SB technology show effectiveness in targeting and killing leukemia cells, providing a potential therapeutic option with a favorable safety profile. This development is significant for advancing CAR T-cell therapies, particularly in improving patient outcomes, while minimizing adverse effects. This technology is also currently applied to allogenic products, while being integrated into closed systems solutions [89], which resulted in the recent initiation of a trial on an allogeneic product targeting CD19 (NCT03389035).

#### 3.2.2. A Mixed AAV-Transposon System to Maximize Yield and Safety

A promising vector technology, recently reported and assessed for its antitumor activity in vivo by a group in Yale, combines mRNA, AAV vector and transposon technologies into one composite system [90]. It has the following two core components: the first is an AAV vector containing the sleeping beauty (SB) transposon comprising the desired transgene. The second is an mRNA encoding the SB100X SB transposase, an enzyme mediating the genomic integration of transposons. The rationale for encoding the transposase in an mRNA is the mRNA’s shorter half-life compared to DNA: if the system used a plasmid DNA, many copies of the transposase could be translated since plasmid DNA is more stable than mRNA [91]. This technique is capable of engineering immune cells efficiently and making the expression of their construct more stable than viral vector methods. Moreover, the frequency of insertions into safe harbors [92], defined as regions of the genome where transgene insertions do not interrupt existing gene activity [93], is higher than lentiviral constructs, as shown by integration profile data from the literature for lentivirus transduction [94], resulting in higher safety. Despite this, estimates of the vector-copy number (VCN) of CAR transgenes per single CAR T-cell [95] were found to be 1–4 copies per cell. This represents a potential limitation of the method, since VCN higher than 1 dose the risk of insertional mutagenesis. Nonetheless, the FDA limit on VCN is 5, making the method still acceptable for commercial applications. Bringing the VCN closest to 1 should be an area of research for this promising manufacturing method.

#### 3.2.3. Using Lipid Nanoparticles as a Contender for Transducing Cars, Engineered for Shorter Production Times

Since 2018, just a year after the FDA approved the first CAR T-cell therapies, another novel manufacturing approach using lipid nanoparticles to reduce production times has been under investigation. One of these investigations reportedly reduced manufacturing time to just 3 days [96]. More recently, even more advanced procedures have emerged, promising to produce a batch of CAR T medicinal products in just 1 day using activating lipid nanoparticles (aLNPs) [97]. These innovative approaches are noteworthy because they enable a scale-up of production that autologous therapies would struggle to achieve by merely increasing volume, particularly when compared to traditional pharmaceutical manufacturing or even heterologous products. Instead of producing more to meet the demand, these methods achieve the same objective by accelerating production, making them an attractive option for patients with rapidly progressing cancers who might otherwise be unable to benefit from CAR T-cell therapy.

These protocols often use closed systems that simplify manufacturing by eliminating bead-mediated T cell activation and reducing external culture. The integration of manufacturing advances and cell engineering reduces production time, setup, and maintenance costs, while enhancing the production capacity of centralized facilities. Recent evidence from the same research group supports the potential to decrease CAR T-cell production time [98].

### 3.3. Open Versus Closed System for Producing ATMPs

The majority of processes used in ATMP manufacturing are *open*, in that the materials, such as cells and reagents, are exposed to the external environment during handling. Conversely, a closed system would keep the entire production process contained within sealed equipment, minimizing exposure to the external environment.

Despite their advantages, closed systems are not widely adopted in ATMP manufacturing due to their inability to handle variability in starting materials and to adapt to unexpected issues during production. Open systems offer greater flexibility during production, allowing operators to make manual adjustments, and resulting in advantageous approaches especially for small-scale or early-stage production, where customization is essential. However, they carry a higher risk of contamination since materials are directly exposed, that require the adoption of strict aseptic techniques and expensive cleanroom environments, with consequent increase in costs and complexity.

Closed systems, on the other hand, encapsulate the production process within controlled environments, often utilizing automated bioreactors. This reduces contamination risks significantly, making them ideal for large-scale production where safety and consistency are critical. They also lower the need for expensive cleanroom setups but tend to lack the flexibility required for complex or individualized ATMPs, and overall have high upfront costs due to the advanced technology involved.

#### 3.3.1. A Closer Look at Closed Systems for Car T

**Figure 3:**
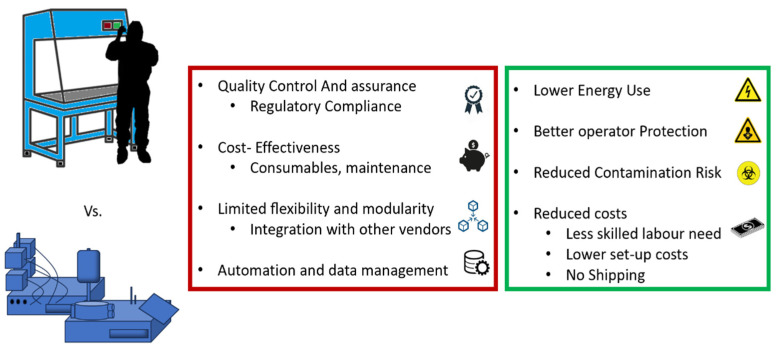
Current limitations and potential benefits of closed systems in ATMP production when compared to open systems adopted by authorized products.

The development of new CAR constructs often starts on a small scale. Heterologous production mimics traditional pharmaceutical manufacturing, but the production of autologous, bespoke therapies requires innovative scaling solutions, typically using closed systems. These systems employ semi-automated machinery and single-use components, isolating the biological material from the operator and offering a cleaner environment comparable to grade A facilities (cleanrooms), even in lower-grade settings. This design minimizes contamination risks and operator exposure to hazardous materials, potentially reducing cleanroom-related costs and logistics [98,99]. These factors contribute to more sustainable operations, reducing energy consumption, easing the burden on operators, and shortening patient wait times. In EU, compliance with GMPs for these automated systems is supported by specific EU guidelines for ATMPs [100,101]. Since adopting distributed manufacturing is crucial for scaling up personalized therapies affordably and supports local production during clinical trials, automation seem to be the correct path as it promotes reproducibility and cost-efficiency by reducing manual steps and the need for specialized workforce and cleanroom facilities, also speeding up the production. However, challenges such as the cost of consumables, equipment incompatibility, and potential supply shortages remain. The limitations of closed systems available on the market versus their potential benefits are illustrated in Figure 3.

#### 3.3.2. Less Is More: The Solution for Larger Production Are Smaller Bioreactors 

Autologous CAR T-cell manufacturing relies heavily on fed-batch and manual processes, which lack environmental monitoring, are prone to variability, and often face issues of contamination [102]. These processes are centralized, complex, and time-consuming, resulting in low production outputs, high costs, and long vein-to-vein times, typically 21–37 days [103]. Key bottlenecks include large equipment footprints, low manufacturing efficiency, and lengthy expansion phases of up to 14 days [104,105], followed by additional time for formulation, cryopreservation, and shipping [106]. Current closed systems, like the CliniMACS Prodigy©, (https://www.miltenyibiotec.com/US-en/products/clinimacs-prodigy.html, accessed on 18 September 2024), improve sterility and consistency but still rely on large volumes and costly reagents, limiting scalability and throughput [107,108]. To address these challenges, decentralized, automated point-of-care manufacturing units and scaled-down perfusion-capable microbioreactors offer potential solutions [109]. These systems, with smaller footprints and working volumes, may overcome the limitations of fed-batch cultures, offering improved efficiency, reduced costs, and greater scalability for autologous CAR T-cell production [110].

A recent study on microfluidic technologies [111] tested the 2 mL Mobius Breez© microbioreactor (Erbi Biosystems, MilliporeSigma, Stoneham, MA, USA) for the activation, transduction, and expansion of human primary T cells to produce anti-CD19 CAR T-cells from patient and donor cells using lentiviral transduction [112]. The device supports cell-culture-on-a-chip [113] and was compared with manual gas-permeable wells in terms of cytokine secretion, persistence, proliferation, and in vivo antileukemic activity. CAR T cells produced in the microbioreactor showed higher glucose and lower lactate levels due to continuous medium circulation, improving nutrient replenishment and waste removal [114]. The microfluidic system generated therapeutic CAR T-cell doses from limited starting materials, using less lentiviral vector (LVV) than the manual method, while achieving comparable transduction efficiency. However, higher proportions of T cells in the microbioreactor expressed differentiation and senescence markers. The device features real-time monitoring of flow rate, dissolved oxygen, and pH, allowing closed-loop control via pneumatic adjustments, ensuring consistent culture conditions [115]. This monitoring could be integrated with computational models for nondestructive assessment of cell health and expansion rates. The Breez system supports parallel production runs across multiple pods, which could reduce labor and cleanroom costs, enhancing decentralized CAR T-cell production. Although the microbioreactor still requires manual handling and biosafety cabinet (BSC) use, future iterations could operate with smaller footprints in noncleanroom environments [116].

In vitro studies showed that CAR T cells from the microbioreactor secreted lower levels of inflammatory cytokines (GM-CSF, IFN-γ, TNF-ɑ), but higher levels of type 2 cytokines (IL-4, IL-5, IL-13) which are linked to long-term remission [117]. In vivo, these cells performed similarly to manually produced CAR T cells in a xenograft NSG mouse model of B-cell leukemia. Future advancements in metabolite sensors, such as Raman spectroscopy [116], could further improve CAR T-cell manufacturing by enabling feedback-driven processes that optimize cell expansion and differentiation [117]. This would lead to more consistent and efficient production, potentially transforming CAR T-cell therapy through adaptive manufacturing systems that respond to donor-specific variability [118,119]. Optimizing factors such as growth media and activation agents [120] will be critical for enhancing cell yields and phenotypes in these systems for clinical applications.

#### 3.3.3. Reports on Real-World Closed Systems Productions

Shalabi H. and colleagues from Stanford [121] reported using on-site, automated, closed-loop manufacturing with a consistent 7–10-day manufacturing turnaround time in two parallel phase 1b clinical trials (NCT04088864 and NCT04088890). They investigated a humanized CD22-CAR with 41BB costimulatory domain in children and adults with heavily treated, relapsed/refractory (r/r) B-ALL, respectively. This development is notable because multicenter trials have been hampered by numerous barriers to adopting a decentralized CAR T manufacturing model. The study demonstrated the feasibility of an alternative automated closed-loop manufacturing approach, which is likely comparable in safety and efficacy to conventionally manufactured products. The safety profile was reported favorable, with only one high grade (3–4) CRS and one immune effector-cell-associated neurotoxicity syndrome (ICANS) occurring throughout the trial. Both side effects occurred in the same patient, who had an ongoing infection, as already reported in authorized products for pediatric and adult r/r B-ALL [122,123]. The high efficacy is attested to by high CR rates in both trials, reached by 75% of patients treated. While these studies also established the feasibility of manufacturing CD22-CARs using the NCI CD22-CAR vector and established a faster time of production than authorized products, duration of response was overall limited (median 77 days), highlighting the need for additional improvements. Patients in these CD22-CAR trials were very heavily pretreated, with a median of six prior lines of therapy. The low rate of post-CAR consolidative hematopoietic cell transplantation (HCT) is likely due to prior HCT in most patients. More fit starting material, either autologous from earlier treatment, or heterologous from off-the-shelf use or iPSC sourcing, may translate in improved survivability. The group also assessed CD22 expression and found that the target was downregulated in a third of available samples at relapse, raising the issue of antigen escape via downregulation or mutation, which has emerged as a key resistance mechanism in CD19-targeted therapies [124]. Notably, three patients developed a side effect not usually associated with CAR T cells, as follows: immune-effector-cell-associated hemophagocytic lymphohistiocytosis-like syndrome (IEC-HS). It has been suggested that this side effect happens regardless of manufacturing method and could be caused by either this CAR construct or by targeting CD22, as CD19 does not result in IEC-HS. CD22-CAR was manufactured using an automated closed CliniMACS Prodigy lentiviral transduction system (Miltenyi Biotec, Bergisch Gladbach, Germany) [125] previously used to successfully create single and dual-targeted CARs [126] at Stanford University or Miltenyi Biotec. The treatment was preceded by a standard lymphodepletion regiment (fludarabine 30 mg and cyclophosphamide 500 mg) followed by fresh or cryopreserved CD22-CAR infusion dosed at 1 × 106 cells/kg (±20%) like in a previous trial [127,128].

#### 3.3.4. Reports on Real-World Heterologous Car T-Cell Productions: The Italian Data

Under hospital exemption (HE), CAR T-cell production in Italy has increased over the past three years. For the treatment of patients with oncologic and onco-hematologic diseases resistant and refractory to major lines of therapy and with reduced lymphocyte fitness, allogeneic CAR T production has emerged as the only therapeutic alternative. Three types of CAR Ts, directed toward GD2, CD19 and CD7 antigens respectively, have been proposed in the HE regimen. All products were obtained from HLA-compatible HSC donors to minimize the risk of developing Graft versus Host Disease (GvHD). GD2 cells, used in neuroblastoma treatment and already included in a phase I/II trial for autologous analogue, are produced in 13 to 14 days by transduction of donor lymphocytes with a retroviral vector expressing GD2 antigen, prepared from the HEK Vec 293 cell line. In contrast, CD7 and CD19 CAR T cells are produced by transduction of donor cells with a lentiviral vector for the treatment of T-cell and B-cell acute lymphoblastic leukemia (T-LLA and B-LLA), respectively. For CD7 CAR T, cells are transduced with a replication-defective lentiviral vector encoding a promoter with an anti-CD7 CAR and anti-CD7 PEBL transgene. Cells directed toward the CD19 antigen are produced by a process involving the use of the automated CliniMACS Prodigy© system that provides a closed environment for transduction of cells with the lentiviral vector encoding for the anti-CD19 scFV, cell expansion and harvesting. Both manufacturing processes last 12 days, then the Drug Product (DP) is frozen and cryopreserved at −70 °C to be freshly infused. For all three types of CAR T produced under HE, to reduce the risk of toxicity, donor cells are engineered to express the gene encoding for inducible caspase iC9 as a safety switch in case of GvHD or toxic responses to treatment. Based on experiences with similar products produced under HE but of autologous origin, the doses selected to treat patients are 1 × 10^6^ cells/kg, 3 × 10^6^ cells/kg, and in only two cases 2 × 10^6^ cells/kg. Data on the outcomes of these therapies, although limited, reveal a fair ability to induce improvement in the clinical setting. Patients treated with allogeneic CAR T cells for B-ALL were able to achieve remission both at the bone-marrow and molecular levels in only three cases. For T-ALL, no follow-up data are currently available for patients treated with allogeneic products. Five deaths occurred for patients treated with GD2 and CD19 CAR T cells, which in none of the cases can be ascribed to the proposed treatments. The deaths recorded are attributable to relapses that occurred after a reduction in circulating CAR T levels or the subsequent development of graft versus host disease, observed in one patient. For patients treated with anti-CD19 and anti-GD2 CAR Ts, follow-up reports revealed adverse drug reactions (ADRs), like grade I/II cytokine release syndromes (CRS) and hematological toxicity (e.g., neutropenia, thrombocytopenia, and anemia). The latter is attributable to the strong lymphodepletion they underwent prior to treatment. Based on promising clinical observations produced under hospital exemption and previous experiences [129], allogeneic CD19 CAR T cells have recently been approved in Italy for a phase I, first-in-man clinical trial targeting pediatric patients with relapsed and refractory B-cell acute lymphoblastic leukemia (Eudract 2023-508420-36-00).

### 3.4. Next Steps and Considerations

Whatever approach is adopted for manufacturing ATMPs, a significant use of consumables is anticipated. The harmonization of consumables, particularly for closed systems, should be a priority for both vendors and regulators. If one-time-use plastics, like piping, bags, and other materials required for the operation of automated systems, were made interchangeable—compatible across various closed-system platforms, regardless of the vendor—this would foster competition, reduce prices, and ensure stable supply and quality standards. Furthermore, creating harmonized standards for closed-system supplies could simplify their use and integration.

These interchangeable components should adhere to standardized designs and measurements in line with general regulations governing closed-system good manufacturing practices (GMPs). Nonetheless, in practice, vendors are unlikely to allow competitors to produce consumables compatible with their proprietary systems, and regulators will continue to require individual production sites to validate their processes, even when using virtually identical items from different vendors.

A potential solution to these barriers to harmonization could be for vendors to preemptively validate substitutes in their systems, so that, in cases of shortages, production could be swiftly switched to a pre-validated alternative component, ensuring continuity. This issue became particularly evident during the COVID-19 pandemic, underscoring the need for vendors to perform comparability exercises with one another under the guidance of competent authorities.

**Figure 4:**
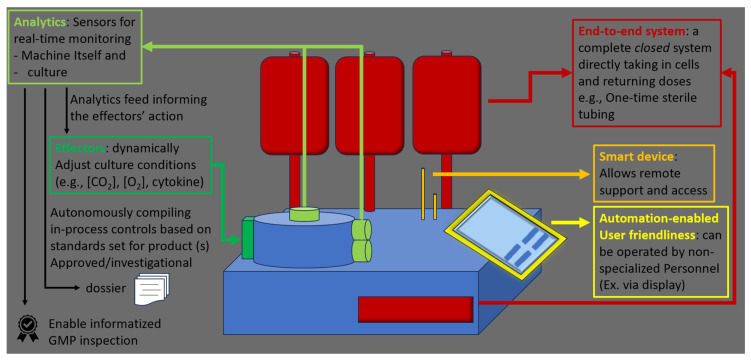
The most useful features of future closed-system devices for expediting production, reducing downtime, and increasing manufacturing quality.

More in general, “GMP-in-a-box” systems enable lower-grade cleanrooms and smaller footprints, allowing smaller centers and developers to manufacture without the upfront cost and regulatory hurdles of setting up A-grade cleanrooms [130]. Although their supply chain may be constrained by reliance on a single vendor, due to the lack of harmonization, these systems generally simplify logistics. To become the leading manufacturing platform for autologous CAR T-cell therapies, future closed systems will likely incorporate AI-enabled technologies. Smart automation products, equipped with sensors that provide real-time data and effectors capable of executing automated responses, will allow systems to adapt to the specific needs of each cell culture. This optimization will not only shorten processing times and reduce material usage but also ease the operator’s workload and minimize the risk of errors. Real-time sensors could also support advanced data management systems, by autonomously conducting in-process quality control and generating validation and batch release data with speed and accuracy. The flexibility of these devices would stem from fine-tuned parameters and algorithms that adjust cell processing based on the features of the starting material. Future systems will also include enhanced cell sorting capabilities to identify and eliminate potentially harmful cells—such as clones that could lead to secondary malignancies—already present in the patient’s blood. While such adaptive manufacturing will require extensive validation, it aligns with the trend in traditional pharmaceutical production, as follows: the more automated the process, the lower the risk of variability caused by human error. Simplifying the manufacturing process will also reduce the need for highly qualified manual interventions. By replacing scientists with technicians and integrating more capabilities into a single closed system, manufacturers can achieve significant cost savings, broaden patient access, and enhance operational resilience, potentially enabling hospitals to maintain miniature GMP factories onsite. The essential features of next-generation closed systems are summarized in Figure 4.

However, comparability validations will be crucial to ensure consistency across new manufacturing units, and if heterologous products achieve the same efficacy as autologous therapies, traditional manufacturing sites separated from the point of care may become more common.

## 4. Conclusions

Current authorized CAR T-cell therapies are autologous and require bespoke manufacturing operations, which result in high costs and limited accessibility. Additionally, their curative efficacy, particularly in solid tumors, remains limited.

This review explores the recent advances aimed at overcoming these challenges. New developments in synthetic biology and genetic engineering offer potential solutions to issues like short-lived responses and antigen escape, while emerging manufacturing methods—such as compact and automated “closed systems”—promise to improve product consistency and reduce labor requirements, thereby shortening production times and lowering costs. However, despite these advancements, autologous manufacturing in open systems remains the standard. This review highlights the significant limitations of these open systems, including their high costs, complexity, and time-intensive processes, which hinder scalability and restrict broader access to treatments. Similarly, while closed systems offer improvements in standardization, they currently lack the flexibility and automation needed to efficiently handle the variability in starting materials, making them less viable in economic and regulatory terms. To address these issues, the review presents potential solutions such as perfusion-capable microfluidics and real-time monitoring technologies, and it points to promising future directions for autologous CAR T manufacturing. Additionally, reports from emerging economies demonstrate that decentralized and point-of-care manufacturing could offer more affordable alternatives. The key takeaway is that no single innovation will resolve all of the limitations of CAR T technology. Instead, the integration of multiple advanced solutions—such as more sophisticated closed systems, transposon-based manufacturing, and gene editing—will pave the way for safer, cheaper, and more accessible CAR T therapies. Achieving effective allogeneic CAR T production could further transform the field, potentially reducing production times and costs to levels like other high-end oncologic drugs, thereby revolutionizing the accessibility and scalability of CAR T-cell therapies, making them economically feasible for National Healthcare Systems.
